# The Influence of Social Support in PROMs of Patients with COPD in Primary Care: A Scoping Review

**DOI:** 10.3390/healthcare11243141

**Published:** 2023-12-11

**Authors:** Antonia Aravantinou-Karlatou, Izolde Bouloukaki, Antonios Christodoulakis, Ioanna Tsiligianni

**Affiliations:** 1 Department of Social Medicine, School of Medicine, University of Crete, Voutes-Stavrakia, 71003 Heraklion, Greece; izolthi@gmail.com (I.B.); christodoulakisa@icloud.com (A.C.); i.tsiligianni@uoc.gr (I.T.); 2 Department of Nursing, School of Health Sciences, Hellenic Mediterranean University, 71410 Heraklion, Greece

**Keywords:** COPD, social support, PROMs, primary care

## Abstract

Chronic obstructive pulmonary disease (COPD) is a prevalent and multidimensional disease with symptoms that greatly influence patients’ health. Healthcare professionals utilize patient-reported outcome measures (PROMs) to classify and better manage the disease. Despite the value of PROMs, they inadequately represent some important dimensions of COPD, like social support and healthcare access/utilization. This is important, especially for social support, since it can positively influence PROMs results and the overall health of patients with COPD. Therefore, a scoping review was conducted to determine how social support affects PROMs of patients with COPD in primary care. The PRISMA–Scoping approach was adopted, and we sought articles published in MEDLINE and COHRANE. We screened 2038 articles for inclusion and finally included a total of 10 articles. Most of the articles were conducted in the U.S. and Norway. Social support had a strong positive impact on PROMs. Additionally, different types of social support were observed. Moreover, higher levels of social support were linked to better quality of life, mental health, self-care behaviors, self-management, functionality, and less severe COPD. Consequently, this scoping review highlights the value of social support in patients with COPD and its underrepresentation and misrepresentation in PROMs literature.

## 1. Introduction

Chronic obstructive pulmonary disease (COPD) is widely accepted as a leading cause of chronic disability, morbidity, and mortality, imposing a major and growing economic and social burden [1,2,3]. It is characterized by a persistent, often progressive obstruction of airflow due to several airway abnormalities (such as bronchitis, bronchiolitis, and emphysema) and chronic respiratory symptoms (e.g., shortness of breath, mucus production, and/or exacerbations) [2]. COPD represents an important preventable, multidimensional, manageable, and treatable public health challenge [2]. Interestingly, to manage and treat patients with COPD, healthcare professionals have to use validated questionnaires to assess quality of life and health status. However, there is a limited association between the severity of airflow obstruction and patient symptoms/health status impairment [2].

Given the aforementioned factors, there is an evident need for validated questionnaires that enable healthcare professionals to assess all dimensions of COPD (e.g., symptoms, physical functioning, psychosocial well-being, etc.) [4,5,6]. For this purpose, several patient-reported outcome measures (PROMs) have been developed and are valuable tools in day-to-day clinical practice [7,8,9,10,11]. These measures are standardized questionnaires that healthcare professionals utilize and to which patients with COPD can respond based on their perception of their health and illness [12]. Through PROMs, healthcare professionals can gain a better understanding of the impact and progression that COPD has on patients and provide better quality of care [7,8,9,10,11,13]. Consequently, by utilizing PROMs, healthcare professionals can address the physical, emotional, and social functioning of COPD patients [4,5,6]. However, despite the value of PROMs, certain dimensions of COPD, such as social support and healthcare access and utilization, remain inadequately represented within these measures [14].

The concept of social support involves the provision of emotional, informational, and instrumental assistance to individuals through their social networks, which includes family, friends, peers, and healthcare professionals [15]. Social support has been classified into two domains: structural and functional. Structural social support encompasses the features of the social network surrounding an individual and their interactions within it (e.g., marital status and living arrangements) [16]. Conversely, functional social support pertains to specific assistance given to an individual through their social network [17]. For patients with COPD, higher levels of social support can play a pivotal role in shaping their experience with the disease and their overall health, potentially influencing the results measured by PROMs [14,18].

Taking into account the effect that adequate levels of social support can have in patients with COPD [19], it is plausible to hypothesize that higher levels of perceived social support may have a positive impact on many PROMs of patients with COPD. However, there is a lack of consistent evidence to support the relationship between perceived social support and self-perceived health in patients with COPD using PROMs, especially in primary care settings. Therefore, a scoping review that explores the interplay between social support and PROMs in primary care patients with COPD could provide valuable insights to the healthcare community. These insights could offer a nuanced understanding of the role of social support in shaping the holistic well-being of primary care patients with COPD. Furthermore, it could contribute to the design of interventions and policies that optimize the patient experience, promote better coping strategies, and improve the overall management of COPD in primary care settings. In light of the aforementioned considerations, the aim of this review was to explore the interplay between social support and PROMs in primary care patients with COPD.

## 2. Materials and Methods

This scoping review was conducted in accordance with the principles recommended in the Preferred Reporting Items for Scoping Reviews (PRISMA-ScR): Checklist and Explanation [20] and the Joanna Briggs Institute Reviewers’ Manual for Scoping Reviews [21].

### 2.1. Search Strategy

Regarding the search strategy, we performed a comprehensive literature search in two electronic biomedical literature databases (MEDLINE and COCHRANE) in September 2023. Since this was a scoping review [22], we wanted to make the literature search as broad as possible. Therefore, we used the following keyword combinations and Boolean operators (AND, OR, NOT) [23] in both databases: “COPD” AND “social support” OR “chronic obstructive pulmonary disease” AND “social” AND “patient-reported outcome measures” OR “PROMs” AND “primary care”.

### 2.2. Study Inclusion and Exclusion Criteria

The titles and abstracts were initially examined for possible inclusion by two independent reviewers. After removing duplicates, the two reviewers collaborated to screen the remaining records. During the screening process, any inconsistencies between the investigators were resolved by a third reviewer.

We included both qualitative and quantitative studies, such as cross-sectional studies, observational studies, interventional trials, longitudinal studies, randomized controlled trials, and qualitative research (e.g., interviews and focus groups). These articles reported on the influence of social support in PROMs in primary care patients with COPD. Additionally, we included articles that examined the influence of various aspects of social support (emotional, instrumental, and informational) in the PROMs of patients with COPD. The articles we included had the full texts available, in the English language, and covered adult patients (18 years and older) that were diagnosed with COPD who received care in primary care settings. It is worth mentioning that the benefits of social support on health have been recognized since 1976 [24]. In addition, this was a scoping review of a relatively understudied topic (the interplay between social support and PROMs in primary care patients with COPD); therefore, we did not specify a timeframe for the inclusion of published studies.

We excluded case reports, case series, commentaries, editorials, letters, conference abstracts, review studies, book chapters, and studies published in languages other than English. Studies involving patients with conditions other than COPD or those conducted in non-primary care settings (e.g., hospital settings and specialized clinics) were also excluded. In addition, gray literature, such as articles that were not peer-reviewed, were excluded.

### 2.3. Data Extraction and Analysis

The methodology employed in this review entailed the extraction of information regarding study design, student population characteristics, and outcomes of interest from the full texts of the included articles by a single reviewer utilizing a standardized data extraction form. Subsequently, the accuracy of the data extraction form was verified by two independent reviewers via a thorough appraisal process, followed by a discussion to resolve any discrepancies. The extracted data were subsequently presented in a descriptive manner in this review. It should be noted that we used the terms “social support”, and “COPD” to identify and include articles, whereas the terms “review”, “cancer”, and “children” were used to identify and exclude articles.

## 3. Results

### 3.1. Trial Flow and Overview of Selected Studies

**Figure 1:**
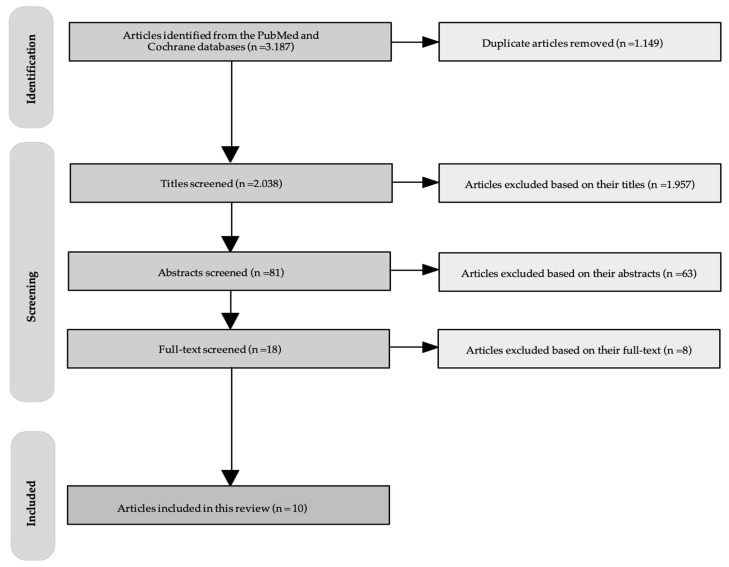
PRISMA flow diagram of the literature.

The initial database search yielded 3187 articles for this scoping review. After the first screening and removal of duplicates, 2038 articles were screened based on their titles. Following that, 81 titles met the inclusion criteria and were selected for a second/further evaluation based on their abstracts. Subsequently, 18 abstracts met the inclusion criteria; therefore the full texts were retrieved for further screening. However, from the 18 full texts retrieved, 8 articles were excluded based on the inclusion/exclusion criteria discussed in the methods section. Therefore, 10 full-text articles were finally included for this review. The PRISMA flow diagram [25] for the literature search is presented in Figure 1.

### 3.2. Characteristics of Included Studies

The characteristics of the 10 included studies are presented in Table 1. Out of the 10 articles, 6 were categorized as cross-sectional, 3 as prospective, and 1 as longitudinal. Regarding the country in which articles took place, most articles were from the U.S. (2 articles) and Norway (2 articles), while the rest came from Australia (1 article), the United Kingdom (1 article), Taiwan (1 article), Spain and Colombia (1 article), Korea (1 article), and China (1 article).

The articles included employed different assessment tools to evaluate social support. In particular, the most common approach was to rely on self-reported questions (4 articles) rather than using validated tools to assess social support. The tools used were the Behavioral Risk Factor Surveillance System (BRFSS) (one question about social support), Medical Outcomes Social Support Scale (MOSSS), Duke–UNC Functional Social Support questionnaire (DUFSS), Multidimensional Scale of Perceived Social Support (MSPSS), European Social Survey (ESS), Illness-Specific Social Support Scale (ISSS), and Social Support Rating Scale.

The PROMs used in patients with COPD were the Hospital Anxiety and Depression Scale (HADS) (2 articles), self-reported questions for COPD (4 articles), modified Medical Research Council Dyspnea Scale (mMRC), and other instruments such as the Behavioral Risk Factor Surveillance System (BRFSS) (3 questions about quality of life); Short Form 12 (SF-12, V2); Living with Chronic Illness Scale (LW-CI Scale); Satisfaction Life Scale (SLS-6); Patient-Based Global Impression of Severity Scale (PGIS); Memorial Symptom Assessment Scale (MSAS); Coping Strategy Indicator (CSI); Functional Performance Inventory—Short Form (FPI-SF); Coping Illness Questionnaire (CWIQ); St. George’s Respiratory Questionnaire (SGRQ); Beck Depression Inventory (BDI); State Trait Anxiety Inventory (Form X-2, trait: anxiety); Control, Autonomy, Self-realization and Pleasure Scale (CASP-19); Chinese version of the 13-item Patient Activation Measure (PAM-13); COPD Assessment Test; and Brief Illness Perception Questionnaire (BIPQ).

### 3.3. Associations between Social Support and PROMs in COPD Patients

Table 1 provides a summary of the main findings from the 10 included articles, examining the correlation between social support and PROMs in patients with COPD.

The general consensus among the articles was that social support has a favorable influence on PROMs. There were three domains in which there was consistent evidence of the positive impact of social support on patients with COPD: mental well-being, quality of life, and self-efficacy.

Four articles [26,27,31,33] were identified that reported a positive impact of social support on mental well-being, specifically on depressive symptoms (2 articles), anxiety (1 article), and psychological well-being (1 article) in patients with COPD. Arabyat et al. indicated in their unadjusted analysis of a large U.S. population-based health survey that a reduced level of social/emotional support was associated with a greater likelihood of experiencing depressive symptoms [26]. Notably, patients lacking sufficient social/emotional support were almost four times as likely to report more than 14 mentally unhealthy days within the past month, in contrast to patients with adequate social/emotional support [26]. Furthermore, in a previous article, increased levels of negative social support were identified as being linked not only to higher levels of depression but also to anxiety symptoms [33]. Patients who received sufficient support from people with whom they had close relationships exhibited a positive correlation with better mental health, although this correlation was no longer present one year after a COPD-specific patient education program [27]. Also, social support was related to better reported general and emotional health and well-being in people [31].

Self-efficacy and self-care behavior, including adherence, showed consistent improvement when participants reported greater social support in six articles [19,28,30,31,32,35]. In a qualitative study, Chen et al. demonstrated a positive association between social support and self-management [28]. In another article, the support of social groups provided mutual trust, support, and increased self-confidence and motivation for self-care [32]. The greater the level of social support received by individuals, the more effective their coping mechanisms became [19]. The Duke–UNC Functional Social Support questionnaire identified social support as a significant factor in the process of living with the illness, as evaluated by the Living with Chronic Illness Scale (LW-CI Scale), which encompasses acceptance, coping, self-management, integration, and adjustment [31]. One additional article [30] found that participants with a spouse or partner as their caregiver had 11 times higher odds of participating in a pulmonary rehabilitation program than those without a caregiver. In the same article, neither structural nor functional support had an impact on adherence to inhaler or nebulizer medications.

The evidence on the relationship between social support and the variables of functional status, quality of life, and self-rated health was inconclusive, and different articles reported different results (8 articles) [19,26,27,28,31,32,33,34]. The research conducted by Arabyat et al. [26] on a community sample of 1.261 patients with COPD revealed a significant correlation between insufficient social/emotional support and disability, as well as impairment in all aspects of health-related quality of life (HRQoL). In the adjusted analysis, patients with COPD who rarely or never received social/emotional support had a higher likelihood of experiencing diminished physical and mental HRQoL days than those who reported receiving sufficient social/emotional support. Despite this, social/emotional support was not significantly associated with disability or general health. However, it is worth highlighting that the assessment of social support was based solely on a single question (“How often do you receive the social/emotional support you need?”). Another article [27] found that among the quality of life of 60 patients with COPD, as assessed by Short Form 12 (SF-12v2) for quality of life (physical and mental components of quality of life), the mental component score was positively associated with social support, which was evaluated through one question (“I think I have enough support from people with whom I have a close relationship.”). This was not the case in another prospective study involving 406 patients with COPD, since poor family and social support score were positively associated with lower quality-of-life scores [34]. The research conducted by Halding et al. showed that the support of social groups and integration into the groups had a positive effect on quality of life [32].

Conversely, a previous article did not find a meaningful correlation between elevated levels of positive or negative social support (as measured by the ISSS) and quality of life, evaluated using the SGRQ [33]. Moreover, no significant correlation was observed between social support (MSPSS) and functional performance (FPI-SF) [19]. Nevertheless, social support was positively associated with better a experience of symptoms in patients with COPD, thereby affecting their functional performance.

## 4. Discussion

This scoping review explored the interplay between social support and PROMs in primary care patients with COPD and elucidated ways in which social support influences the multi-faceted dimensions of COPD-related well-being. The 10 articles included in this review indicate that social support has a strong positive influence on various health-related PROMs of COPD. Additionally, higher levels of social support were related to better quality of life, self-care behaviors, and self-management in patients with COPD in primary care settings. Interestingly, social support was positively associated with better mental health (anxiety and depression), which in turn was associated with better quality of life and lower severity of symptoms in patients with COPD. In addition, social support was positively associated with less severe COPD and better functional performance in patients with COPD. Simultaneously, another finding was that, despite the clear definition of social support and its domains (structural and functional), researchers measured social support through family, friends, health professionals, and social groups.

A major finding of the present review was that social support could help alleviate multiple health-related problems that patients with COPD experience, such as physical, psychological, and financial burden and stress. Furthermore, social support could have a positive effect on motivation for self-management and adherence to treatment in patients with COPD [36]. This effect could help improve the dyspnea, anxiety, depression, and overall health status and prevent disease deterioration in patients with COPD, as shown in other diseases, such as silicosis [37]. Additionally, patients with COPD described feelings of social isolation and reported suffering from negative emotions [38]. Their personal integrity and self-esteem were threatened due to their dependence on others and their self-blame for the disability inflicted by their condition, which is mainly caused by smoking [38,39,40,41]. A potential explanation for the positive effect that social support has on patients with COPD was that it serves as a protective mechanism during stressful life events, such as COPD diagnosis/exacerbations [42]. This means that social support could act as a barrier to mitigate the negative effects of COPD on patients [43]. Moreover, the impact of stressful situations could be more significant for individuals who feel that they receive lower levels of social support than those who feel that they receive higher levels of social support [42,43]. In addition, social support may have the potential to improve the coping mechanisms of patients with COPD by boosting their problem-solving skills, enhancing their comprehension of the disease, and fostering increased motivation to take action [44].

Patients with other chronic diseases, such as diabetes, chronic heart disease, and chronic kidney disease, have also been found to exhibit a connection between improved self-care behaviors and increased levels of social support [45,46,47]. However, there have been only a handful of articles on patients with COPD that have examined the relationship between social support and self-care behaviors. For example, two articles revealed that participants with COPD were able to better manage their condition when they received functional social support from their family members [48,49]. A potential explanation for this finding could be that having sufficient social and emotional support can directly improve mental health regardless of whether one is facing stressful situations [43]. Moreover, individuals with greater levels of social support tend to experience increased self-esteem, a sense of security, and better decision-making when it comes to healthcare [43]. Indeed, studies have emphasized the relationship between social support and its potential impact on stress-related conditions such as COPD [50,51]. Specifically, research has suggested that greater levels of social support may lead to a reduction in psychological problems and a more rapid recovery from stressors, including COPD exacerbations [50,51]. Additionally, higher social support has been associated with a decrease in severe and disabling COPD exacerbations [50,51]. However, the majority of these studies have not investigated the connection between the structural and functional aspects of social support and the performance of self-care activities among patients with COPD. Evidently, there is a great discrepancy in the terminology of social support. For example, almost all studies included in this review defined social support differently (family, friends, health professionals, and social groups), with only one notable exception [30] that measured both domains of social support (structural and functional) [16] by using two different measures. This means that there is an urgent need to better inform healthcare professionals about social support and its dimensions and to decide on common and standardized [52] terminology between different healthcare professionals.

The international and regional guidelines for the management of COPD could concentrate on all aspects of social support and their implementation in patients with COPD, since this has not been highlighted enough. The articles examined in this review not only highlighted the relationship between social support and PROMs but also emphasized the benefits of social support in the overall health of patients with COPD. However, worldwide, there is limited evidence on the influence of social support in COPD. The included articles focused either on pharmacological and medical aspects (symptoms) related to COPD or on non-pharmacological aspects mainly related to exercise or mental health. Additionally, another finding of our review was that the measures and tools used to evaluate social support are not uniform and vary widely across studies. In particular, the majority used self-reported questions and non-validated tools to assess social support. It should be noted that the CCQ [7], a broadly used PROM for COPD, includes one question about an aspect of social support (social activities such as talking, being with children, visiting friends/relatives). However, although the CCQ is included in the Global Initiative for Chronic Obstructive Lung Disease (GOLD) as a suggestion, only the mMRC and CAT are suggested as PROMs to classify patients [2]. Therefore, more efforts are needed to establish social support as an important indicator of PROMs in patients with COPD.

As previously stated, patients with COPD encounter a multitude of challenges in managing their condition, such as breathlessness, fatigue, anxiety, and the social burden it entails [1,2,3]. Primary care plays a crucial role in the diagnosis and management of COPD by monitoring disease progression, exacerbations and medication adherence, and by developing individual action plans [53]. With the aim of the better diagnosis and management of COPD, primary care should utilize PROMs [53]. Conversely, primary care could foster social support for patients with COPD by offering emotional, educational, and informational support through a social network that includes healthcare professionals, caregivers, family, and friends [26,27,33]. Evidently, patients with COPD could benefit greatly from social support, as it can assist them in managing their illness and improving their overall health [26,27,33]. Consequently, the incorporation of social support and PROMs into everyday primary care for COPD patients holds the potential to greatly enhance their overall health and, consequently, their quality of life. It should be noted that healthcare professionals must possess a comprehensive understanding of the daily lives and influencing factors of individuals with long-term conditions (LTCs) to deliver thorough, personalized, and patient-centric care [54,55]. For example, understanding the determinants of living with LTCs, specifically from the individual’s perspective, is an underrepresented topic in the literature. This is important, since two major outcomes of the complex experience of living with LTCs are quality of life and satisfaction with life [55].

The strength of this scoping review was its comprehensive approach to social support and PROMs in patients with COPD. Social support has been associated with improved self-management [56] and general self-efficacy in patients with COPD [27]. These factors have been positively associated with the functionality of patients [57].

Based on these findings, this scoping review provides opportunities for future research. First, we propose that there is a need for validated tools for social support and that further articles are needed. Second, more research should be conducted using a comprehensive approach to clarify the potential causal contributions of social support to patients with COPD. Fourth, guidelines are single-disease oriented and usually approach a chronic condition like COPD by focusing mainly on diagnosis and management, despite the fact that they should approach it multidimensionally (e.g., social and psychological dimensions, health determinants, frailty, multimorbidity, etc.).

### Limitations

Despite the useful findings, the present scoping review is subject to a few notable limitations. First, we included only articles in the English language, therefore limiting our results. Second, PROMs include many different questionnaires, thus making it difficult to compare articles. Third, the definition of social support differed greatly between studies; thus, further analysis/comparisons were difficult. Finally, we did not evaluate the quality of the articles and simply described their findings without further analysis.

## 5. Conclusions

The results of this review show that social support is positively associated with mental health, quality of life, and self-efficacy in patients with COPD. Specifically, higher levels of social support were associated with lower levels of depressive symptoms and better self-care behaviors (adherence) and self-management in patients with COPD. Furthermore, it should be noted that the majority of research pertaining to patient-reported outcome measures (PROMs) in patients with COPD has overlooked the significance of social support. Additionally, there is a general misrepresentation of social support regarding its definition and domains in the current literature. Given this insufficiency in the current literature, it is crucial that future research focus on the significance of social support in PROMs in primary care patients with COPD. Consequently, our review emphasizes the vital role of social workers in the multidisciplinary health team of COPD patients and social support as one of the cornerstones of holistic care for them. Therefore, healthcare managers could aim to provide higher levels of social support in order to improve the quality of life, mental health, and self-efficacy of patients with COPD.

## Figures and Tables

**Table 1:** Articles investigating the impact of social support on COPD PROMs.

Author/Date	Study Type	Setting	Social Support Measure	PROMs Measure	Main Findings
Arabyat et al. (2019) [26]	Cross-sectional	*n* = 1.261 participants (U.S.)	Behavioral Risk Factor Surveillance System (BRFSS); the domain of one question: “How often do you get the social/emotional support you need?”	Behavioral Risk Factor Surveillance System (BRFSS); three questions about health-related quality of life	Over one-third of COPD patients reported rarely/never receiving social/emotional support. COPD patients who were not adequately supported socially/emotionally were significantly different in baseline characteristics in comparison to those who received sufficient support. COPD patients who expressed a lack of social/emotional support were at a higher risk of experiencing physical and mental health issues, including depression, whereas adequate social support was associated with decreased depressive symptoms. Inadequate social/emotional support was found to be associated ** with a decline in HRQoL and an increased likelihood of experiencing depression and disability.
Bonsaksen et al. (2014) [27]	Prospective	*n* = 60 participants (Norway)	Participant response to one question: “I think I have enough support from people with whom I have a close relationship.”	Short Form 12, version 2 (SF-12v2) for quality of life (physical component summary scores (PCS) and mental component summary scores (MCS)). Brief Illness Perception Questionnaire (BIPQ) for illness perception.	Initial findings showed a positive correlation between higher social support and higher MCS scores at baseline. However, this correlation *** no longer existed 1 year after the patient education program. Social support did not mediate the correlations * between illness perceptions and HRQoL.
Chen et al. (2016) [28]	Cross-sectional	*n* = 19 participants (Taiwan)	Qualitative method through in-depth interviews. The topics included questions about social support.	Qualitative method through in-depth interviews. The topics included questions about experience of illness and psychological status.	Patients indicated being provided with positive support from both their family members and healthcare professionals. Thematic analysis based on Miles and Huberman’s (1994) [29] guidelines was performed and showed that social support had a significant effect on COPD self-management. Factors including physical and psychological well-being, disease-related cognition, and social support influenced the self-management efficacy of COPD participants.
Chen et al. (2017) [30]	Longitudinal	*n* = 282 participants (USA)	Participant response to four questions: (1) whether participants live alone or live with others, (2) whether they are partnered, (3) the number of close friends and relatives they have, and (4) the presence of a family/friend caregiver (“Which family member or friend is most involved in your care now?”) for structural social support. Medical Outcomes Social Support Scale (MOSSS) for functional social support.	Hospital Anxiety and Depression Scale (HADS) for psychological symptoms. Participants’ response to four questions about carelessness, forgetting, stopping medication when feeling better, and using less of the medication than prescribed when feeling better in the past 3 months for adherence to inhaler.	High levels of structural and functional social support, as the majority had a supportive environment. Participants with a spouse or partner as their caregiver had 11 times greater odds ** of participating in pulmonary rehabilitation compared to those without a caregiver. Neither structural nor functional support appeared to have * any impact on adherence to inhaler.
Corchon et al. (2021) [31]	Cross-sectional	*n* = 1.788 participants (Spain and Colombia)	Duke-UNC Functional Social Support questionnaire (DUFSS) for perceived functional social support.	Living with Chronic Illness Scale (LW-CI Scale) for complex process of living with long-term conditions (LTC). Satisfaction Life Scale (SLS-6) for satisfaction with life during the process of living with an LTC. Patient-Based Global Impression of Severity Scale (PGIS) for self-perception of disease severity.	Satisfaction with life and social support were highlighted as key contributors to the overall experience of individuals living with LTCs, such as COPD patients. There was a positive correlation * between social support and improved general and emotional health, as well as overall well-being.
Halding et al. (2010) [32]	Prospective-interventional	*n* = 18 participants (Norway)	Qualitative method through in-depth interviews. The topics included questions about social support. Participants responded to questions regarding family life, sources for support, experiences from contact with peers in the last year, and how the participant perceives current everyday life.	Participants responded to questions about experiences in everyday life with COPD prior to pulmonary rehabilitation, symptoms, problems, impact on everyday activities, and psychosocial changes associated with the illness.	The participants emphasized that social integration in rehabilitation groups and support from peers and health-care personnel are important dimensions regarding pulmonary rehabilitation (PR). The support of social groups encouraged mutual trust, support, increased self-confidence, and motivation for self-care. The support of social groups and integration in those groups had a positive effect * on quality of life. The support provided by health professionals relieved the patients’ symptoms.
Jun et al. (2023) [19]	Cross-sectional	*n* = 202 participants (Korea)	Multidimensional Scale of Perceived Social Support (MSPSS) for social support.	Memorial Symptom Assessment Scale (MSAS) for experience of symptoms. Coping Strategy Indicator (CSI) for coping. Functional Performance Inventory—Short Form (FPI-SF) for functional performance.	High levels of social support were associated * with a decrease in experiencing symptom. The more social support individuals received, the better their coping mechanisms were. Higher levels of social support were significantly associated * with lower symptom experience and higher functional performance.
McCathie et al. (2002) [33]	Cross-sectional	*n* = 92 participants (Australia)	Illness-Specific Social Support Scale (ISSS) for social support.	Coping Illness Questionnaire (CWIQ) for coping. Beck Depression Inventory (BDI) for depression. State Trait Anxiety Inventory for anxiety. St. George’s Respiratory Questionnaire (SGRQ) for quality of life.	Positive social support was identified as a factor contributing * to decreased levels of depression and anxiety, whereas negative social support was identified as a factor contributing * to increased levels of depression and anxiety. No significant relationship * was found between high levels of positive or negative social support and quality of life.
Sarwar et al. (2021) [34]	Prospective cohort	*n* = 406 participants (United Kingdom)	Socio-demographic characteristics included questions about family support.	Participant response to questions about self-reported general health. Control, autonomy, self-realization (CASP-19) for quality of life.	Poor family and social support were found to be significantly correlated with a decrease in QoL score. The traits of depression and poor family and social support had the most pronounced impact * on the decline in QoL.
Tang et al. (2022) [35]	Cross-sectional	*n* = 170 participants (China)	Social Support Rating Scale for social support.	Patient Activation Measure (PAM-13) for patient activation. Brief Illness Perception Questionnaire (BIPQ) for illness perception. Hospital Anxiety and Depression Scale (HADS) for anxiety and depression. COPD Assessment Test (CAT) for health status.	Social support demonstrated a strong and positive connection * to patient activation.

* Beta coefficients (β); ** odds ratio; *** univariate correlation coefficients (Pearson’s r).

## Data Availability

No new data were created or analyzed in this article. Data sharing is not applicable to this article.
